# Au–Ag Alloy Nanocorals with Optimal Broadband Absorption for Sunlight-Driven Thermoplasmonic Applications

**DOI:** 10.1021/acsami.2c05983

**Published:** 2022-06-17

**Authors:** Federico Pini, Roberto Pilot, Gloria Ischia, Stefano Agnoli, Vincenzo Amendola

**Affiliations:** † Department of Chemical Sciences, University of Padova, via Marzolo 1, 35131 Padova, Italy; ‡ Consorzio INSTM, via G. Giusti 9, 50121 Firenze, Italy; § Department of Industrial Engineering, University of Trento, Via Sommarive 9, 38123 Trento, Italy

**Keywords:** Au nanoparticles, Ag nanoparticles, AuAg alloy, photothermal effects, sunlight conversion, plasmonics, thermoplasmonics

## Abstract

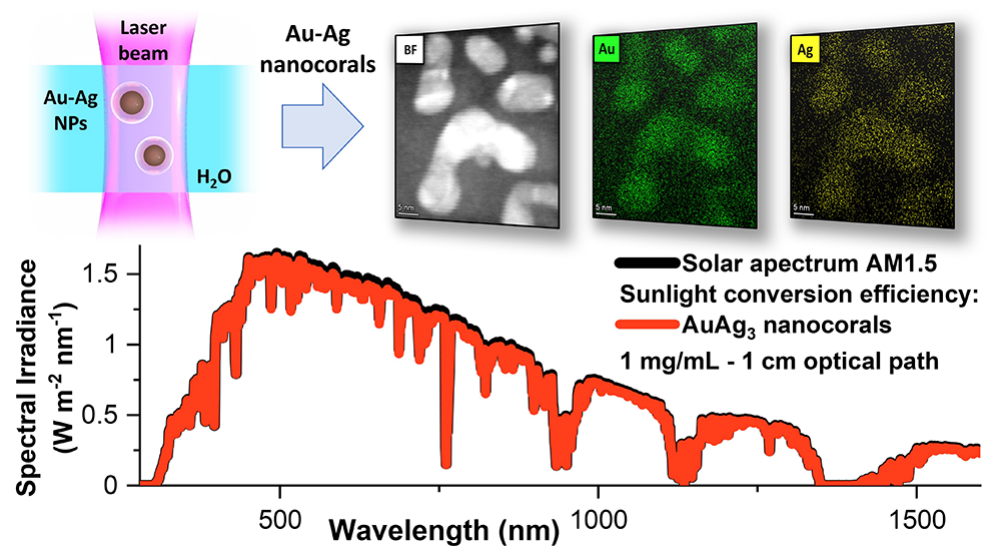

Noble metal nanoparticles are efficient converters of light into heat but typically cover a limited spectral range or have intense light scattering, resulting in unsuited for broadband thermoplasmonic applications and sunlight-driven heat generation. Here, Au–Ag alloy nanoparticles were deliberately molded with an irregular nanocoral (NC) shape to obtain broadband plasmon absorption from the visible to the near-infrared yet at a lower cost compared to pure Au nanostructures. The Au–Ag NCs are produced through a green and scalable methodology that relies on pulsed laser fragmentation in a liquid, without chemicals or capping molecules, leaving the particles surface free for conjugation with thiolated molecules and enabling full processability and easy inclusion in various matrixes. Numerical calculations showed that panchromism, i.e., the occurrence of a broadband absorption from the visible to the near-infrared region, is due to the special morphology of Au–Ag alloy NCs and consists of a purely absorptive behavior superior to monometallic Au or Ag NCs. The thermoplasmonic properties were assessed by multiwavelength light-to-heat conversion experiments and exploited for the realization of a cellulose-based solar-steam generation device with low-cost, simple design but competitive performances. Overall, here it is shown how laser light can be used to harvest solar light. Besides, the optimized broadband plasmon absorption, the green synthetic procedure, and the other set of positive features for thermoplasmonic applications of Au–Ag NCs will contribute to the development of environmentally friendly devices of practical utility in a sustainable world.

## 1 Introduction

In the past decades, noble metal nanoparticles (NPs) have been the subject of extended investigations concerning their intense and tunable localized surface plasmon properties, i.e., the possibility to collectively excite conduction electrons with photons.^1−5^ Among the multiple phenomena and proposed applications of plasmons, the conversion of light into heat, also referred to as thermoplasmonics,^3,4^ is attracting a special interest for the panel of original solutions offered in the field of sustainability and green processes.^3,6^ For instance, thermoplasmonic effects were successfully applied to sunlight-to-heat conversion for steam generation, distillation, desalination, and wastewater treatment.^7−12^ Besides, thermal catalysis of endothermic chemical reactions^7^ has been demonstrated thanks to the efficient and localized heat generation allowed by noble metal NPs.^3^ Sunlight-activated thermoelectric systems have been also proposed.^3^

Considering that, at Earth’s ground (AM 1.5), the 87.7% of sun energy is comprised in the 350–1350 nm range, with the 52.4% at wavelengths >700 nm,^13,14^ a key point is that the plasmonic nanostructures for light-to-heat conversion should cover such a wide spectral range.^3,13,15^ More in general, the list of photothermal applications benefiting of broadband plasmon absorption also extends to the biomedical field, where light-triggered heating in the near-infrared (NIR) biological transparency window I (700–900 nm) or II (1000–1700 nm)^16^ has been used for photothermal therapy,^17^ photoacoustic imaging,^17^ controlled drug release,^18^ and antimicrobial systems.^19^ Besides, thermoplasmonic effects exploited for triggering chemical processes in self-healing materials,^20^ shape-morphing systems,^21^ and photothermal polymerization^3,22^ preferentially rely on red or NIR light to avoid photodegradation and photoionization of the molecular constituents.

Unfortunately, the plasmon resonances of spherical or rod NPs are narrow and centered at specific wavelengths, which is not optimal for harvesting of solar energy,^15,23−26^ even for the most effective plasmon heaters such as nanodoughnut.^27^ Symmetry reduction allows for tuning the number, position, and intensity of plasmons, which become broader and cover a wide spectral range when also the size of the nanostructure is increased over tens of nanometers.^1−3,7,11,28^ Alternatively, new broad resonances from the visible to the NIR arise in large aggregates of NPs due to the mutual coupling of plasmon modes of the neighboring particles.^2,7,18,26^ In particular, several elongated or asymmetric networks of noble metal nanoparticles have been described for their multimodal plasmonic responses extending in the red and NIR.^17,29−33^ However, the light-scattering component scales with the sixth power of object size and rapidly equals or overwhelms the absorption component in large objects, with a consequent loss of photothermal efficiency in big NPs or their aggregates.^11,23,24,34^

Hence, the use of noble metal NPs for sunlight-to-heat conversion requires some key enabling features^3,11,13,15^ like (i) panchromatic absorption, i.e., a broadband absorption from visible to NIR; (ii) minimization of light-scattering and reflectivity; (iii) photostability without reshaping or coalescence during or after operation; (iv) stability in liquid solution for processing and inclusion in nanocomposite matrixes or substrates; (v) easy grafting of chemical components with specific functions for each photothermal application or for optimal integration on each substrate and matrix; (vi) clean surface of the NPs as well as absence of toxic or pollutant residuals as required for catalytic applications (also mandatory in case of biological uses); (vii) limited cost of materials and production, as well as sustainable and scalable synthesis. The last four features are indispensable for marking the advantage of noble metal NPs compared to other absorbers with limited processability, costly functionalization, surface contamination, or lack of scalability of the synthetic protocols.^3,9,13,25,35^

A previous work showed that several of the above criteria can be satisfied by Au nanocorals (NCs) produced with a convenient laser-assisted procedure under continuous flow, in an environmentally friendly way and without chemicals, stabilizers, or templating molecules.^36^ Au NCs have a variety of highly asymmetric elongated shapes with a thin (<10 nm) cross sectional size, supporting multiple low-energy surface plasmon modes in the NIR in addition to normal energy resonances in the visible range.^36^ This overall resulted in a “black” nanogold formulation with a broadband plasmon absorption. However, the absorption cross section of Au NCs is not optimal for sunlight harvesting, due to the prevalence of gold interband transitions below 400 nm. Conversely, silver NPs are known to provide better plasmonic properties than gold, due to a negligible overlap with interband transitions, which is qualitatively evident from the fact that the plasmon absorption bands of Ag NPs are more intense than the interband transitions edge in optical absorption spectra.^2,3,37,38^ Quantitatively, in the visible range, this corresponds to extinction cross sections >3 times larger than Au NPs with the same geometry.^3,38^ Besides, Au has a high cost, making gold nanostructures practically exploitable only for high-value added specific applications, such as in the biomedical field.^1,35,39^ Silver is ca. 75 times less expensive than Au per unit gram, and ca. 140 times less expensive per unit molar volume (equal for Ag and Au), which is the relevant quantity when comparing plasmonic properties, because it determines the free electron density.^2,40^ Although Ag nanostructures have inferior chemical stability than Au,^37,41,42^ it has been shown that alloying Ag with 10–20 at% of Au dramatically improves the resistance even in harsh chemical conditions,^41,43,44^ thanks to surface Au segregation and passivation.^45,46^ In fact, the alloying of metals provides several opportunities for tuning and optimizing materials properties along the desired applicative direction.^12,26,41,45−47^ In the field of plasmonics, for instance, Au–Ag^48^ and Ag–Al^49^ nanoalloys were exploited for tunable surface enhanced Raman scattering substrates and the study of plasmon enhanced catalytic processes. This is also pushing to the continuous development and study of new alloys such as Ag–Cu^50^ or Au–Sn.^51^

Driven by the above considerations, here, we operated to achieve Au–Ag alloy NCs with the plasmonic quality factor and cost-affordability of Ag as well as the compatibility with the self-standing, green and scalable laser-assisted synthetic procedure previously established with Au NCs. Laser irradiation lets metal particles spontaneously undergo a preferential unidirectional growth in solution, without external chemical agents or capping molecules, as a consequence of the balance between the electrostatic repulsion force and the attractive dipolar interactions in the colloidal system,^33,36,52,53^ and the resulting Au–Ag alloy NCs have optimized broadband absorption for sunlight-driven thermoplasmonic applications. Numerical calculations elucidated the correlation of NCs morphology with the observed panchromism, also quantifying the predominance of the absorption contribution over scattering. The thermoplasmonic properties were assessed in different light-to-heat conversion experiments and specifically applied to the realization of a proof-of-concept solar-steam generation device. The results clearly evidenced the set of positive features of Au–Ag NCs for thermoplasmonic applications, which make them utilizable for a variety of environmentally friendly devices of practical utility in a sustainable world.

## 2 Results and Discussion

### 2.1 Laser-Assisted Synthesis

**Figure 1:**
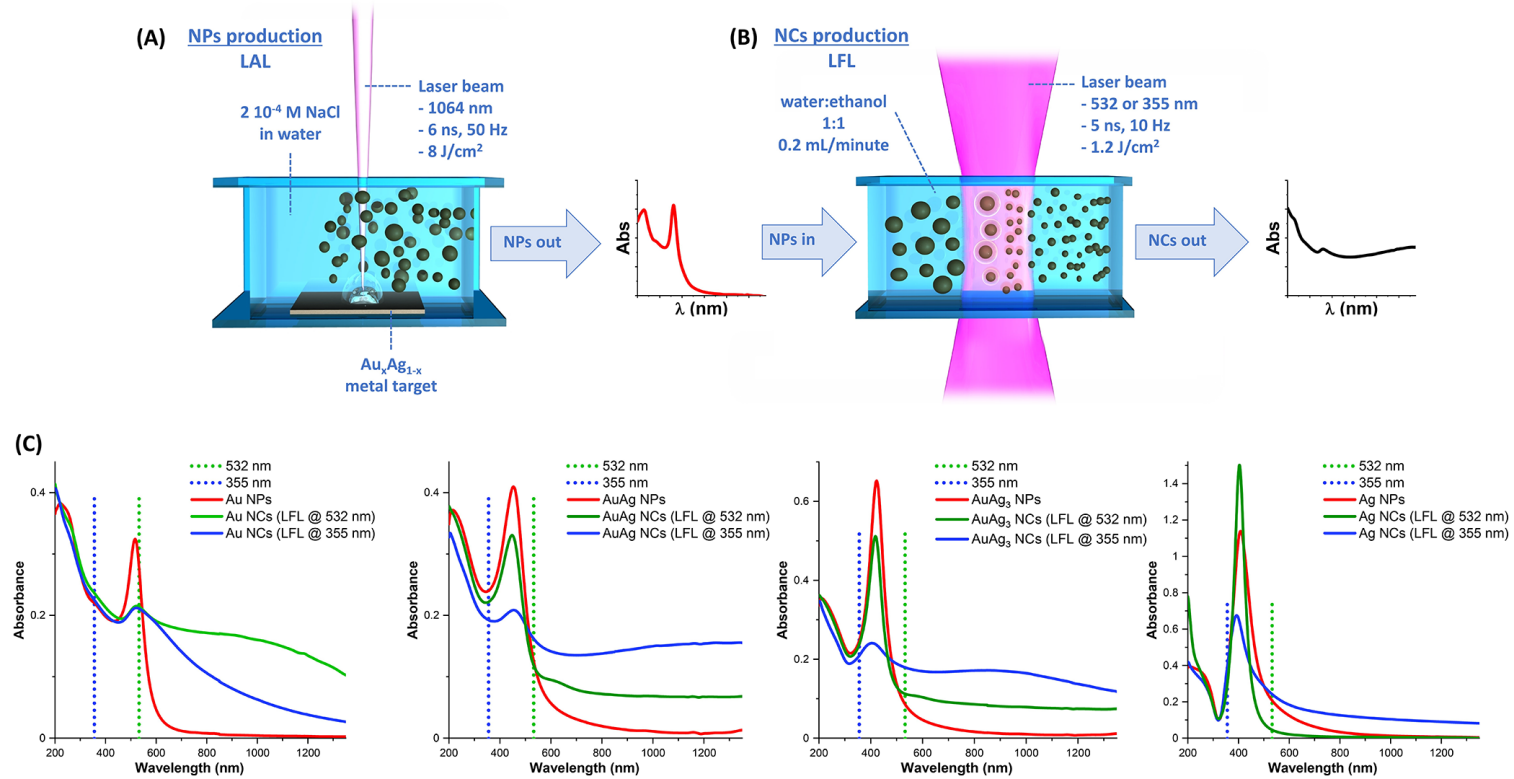
(A and B) Sketch of the laser-synthesis procedure consisting of LAL (A) and LFL (B). (C) UV–vis spectra of pristine Au, AuAg, AuAg_3_, and Ag NPs (obtained from LAL, red lines) and of the corresponding NCs obtained just after LFL @ 532 nm (green lines) or 355 nm (blue lines). Irradiation wavelengths are indicated by vertical dashed lines.

The NCs were obtained in two consecutive steps consisting in the production of colloidal NPs by laser ablation in liquid (LAL, Figure 1A) followed by laser fragmentation in liquid (LFL, Figure 1B) to transform the pristine metal NPs into the NCs. Briefly, in the LAL synthesis, a metal target with the same composition desired for the NPs (Au; Au(0.5)Ag(0.5) alloy, namely, AuAg; Au(0.25)Ag(0.75), namely, AuAg_3_; Ag) was dipped in aqueous NaCl solution (2 × 10^–4^ M) and ablated with NIR laser pulses (1064 nm, 6 ns). The resulting aqueous colloid was mixed 1:1 with pure ethanol at a final metal atoms concentration of 0.5–0.6 mM and injected into a glass tube (1.5 mm diameter) at a flux of 0.2 mL/min, in which the LFL was performed with either 532 or 355 nm focused laser pulses (5 ns). The water/ethanol mixture was selected considering that a higher ethanol content resulted in lower stability of the colloid and loss of material, while at a lower ethanol fraction, the colloidal stability increased at the expenses of the preferential unidirectional assembly of photofragmented metal particles into the coral morphology. Except for NaCl and the two pure liquids, no other chemicals or capping molecules are used in the whole synthetic procedure. Noticeably, LAL and LFL both are self-running processes, and we envisage the possibility to run simultaneously these two steps in a dedicated set up for continuous NCs production, even by remote control.^54^ Besides, the LFL environment requires only water and ethanol, which are class 3 solvents that can be implemented in sustainable production processes. However, we verified that the Au–Ag NCs formation effectively takes place also by LFL at 355 nm in aqueous solution without ethanol, thus avoiding the use of an additional nonaqueous solvent.

The laser wavelength resulted to be a crucial parameter for NCs synthesis, because only the pristine Au NPs have appreciable plasmon absorbance at 532 nm, while the resonance of Au–Ag and Ag NPs is progressively blue-shifted toward 400 nm in relation to the silver content^46,47^ (red lines in Figure 1C). Consequently, the laser irradiation at 532 nm produced a limited (Au–Ag alloys) or null (Ag) photofragmentation, except for Au NPs, which showed a remarkable broadband absorption after LFL (green lines in Figure 1C). More in detail, the plasmon resonance of Ag NCs became sharper after irradiation at 532 nm, and more intense than before the treatment. This is indicative of the reshaping of asymmetric Ag NPs and their aggregates into compact spherical particles.^42,55^ The plasmon absorption of Au–Ag alloy NCs was less intense after irradiation at 532 nm, and a broadband absorption background appeared. While both these features indicate photofragmentation into smaller particles and NCs formation, the effect is much limited compared to Au NCs. Note that a fluence higher than 1.2 J/cm^2^ was avoided because it would result in damaging of the glass tube over time.

At 355 nm, all metal NPs absorb light due to either plasmon or interband electronic transitions, undergoing to photofragmentation.^56^ However, only the Au–Ag alloys exhibit an appreciable new broadband optical absorption typical of the anisotropic regrowth into “coral-like” structures (blue lines in Figure 1C). In the Au NCs sample, a damping of the plasmon resonance, typical of size reduction, is accompanied by a limited broadening of the plasmon absorption. The Ag NPs are halfway between Au and Au–Ag NCs, with a damped plasmon peak still prevailing on the broadband absorption component.

**Figure 2:**
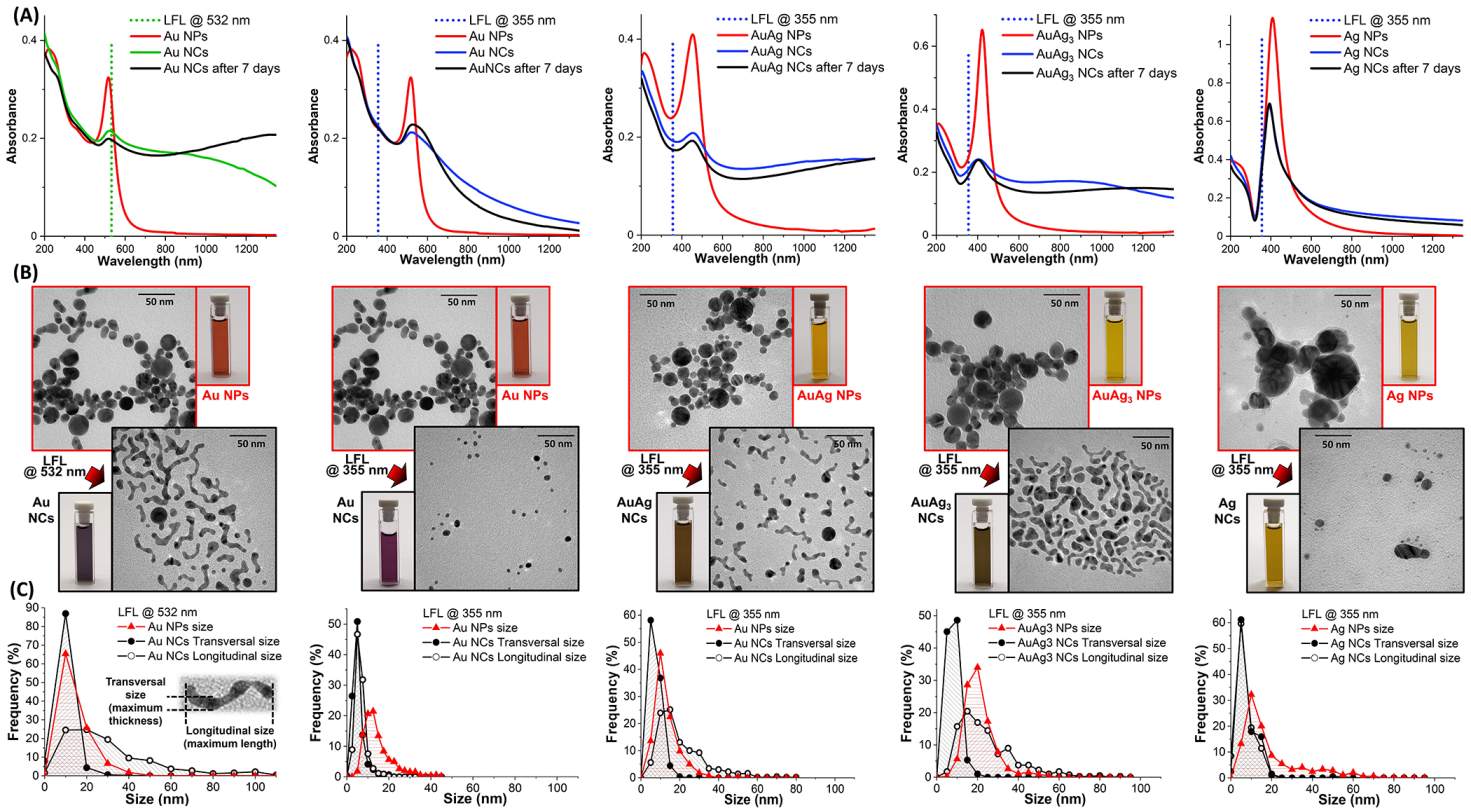
(A) UV–vis spectra of NCs after aging for 7 days in the dark at room temperature (black lines), compared to the spectra of pristine NPs (red lines) and NCs just after LFL (LFL @ 532 nm, green line; LFL @ 355 nm, blue lines). (B) TEM images of NPs before and after LFL, aging for 7 days, coating with PEG and dialyisis. Pictures of the corresponding colloids are also shown. (C) Histograms of size distribution for pristine NPs (red triangles) and aged NCs (transversal size, full black circles; longitudinal size, hollow black circles).

According to our previous study about Au NCs obtained by LFL at 532 nm,^36^ aging over 1 week is associated with a further growth of anisotropic structures and consequent increase of the broadband absorption in the NIR. Hence, the UV–vis spectra were collected after storing the NCs solutions at room temperature in the dark for 7 days, but the results confirmed the main optical features observed just after the LFL. More in detail, the spectra of Au NCs obtained by LFL at 532 nm and Au–Ag NCs obtained by LFL at 355 nm exhibited a moderate increase of the broadband absorption. On the contrary, the spectra of Au and Ag NCs obtained by LFL at 355 nm did not show any appreciable increment of panchromism (Figure 2A).

### 2.2 Structural Characterization

The relationship between optical properties and structure of NCs was investigated further by transmission electron microscopy (TEM). All samples (Au NCs from LFL at 532 nm, Au, AuAg, AuAg_3_, and Ag NCs from LFL at 355 nm) showed a size reduction compared to the pristine spherical NPs (Figure 2B). However, elongated anisotropic morphologies are found only in Au NCs from LFL @ 532 nm, AuAg and AuAg_3_ NCs. In the Ag NCs sample, a few large agglomerates were also found, which cannot be spotted from dimensional distribution analysis, because of their small number compared to the small nanoparticles, but which are expected to contribute to the optical spectrum due to their large volume. In the Au NCs sample obtained by LFL at 355 nm, groups of small sized spheroidal particles are found, without any evidence of NC shapes, explaining the lack of broadband absorption in the UV–vis spectrum. Note that pristine NPs were deposited on the TEM grid directly from the solution used for LFL, without any additive or treatment, while NCs solutions were conjugated with thiol terminated methoxy poly(ethylene glycol) (m-PEG-SH) in order to freeze the NC morphology and avoid coalescence, reshaping, or other effects during solvent evaporation on the TEM grid. In the TEM images, this is well-appreciable by the agglomeration of pristine NPs as opposed to the interparticle separation of NCs.

The structural changes undergone after LFL and aging are evident by the naked eye from the color change of educt NPs and corresponding NCs, especially for the Au–Ag and Au (LFL at 532 nm) samples (see pictures in Figure 2B). These morphological features were transformed into measurable quantities such as the histograms of the maximum transversal cross section of the NCs and their longitudinal length (see the example reported in Figure 2C). By comparison with the size distribution of pristine spherical NPs, it is evident that the transversal size of NCs is systematically smaller. Besides, in the NCs with appreciable broadband absorption, the longitudinal size extends well beyond the transversal size and, in part, also beyond the diameter of the initial NPs. This is a clear indication that, after photofragmentation, the small metal particles preferentially regrew in a unidirectional way, as a consequence of the balance between the attractive dipolar interactions in the colloidal system and the electrostatic repulsion forces, which are weaker along the axis of an elongated particle (e.g., after sticking of two nanospheres).^33,36,52,53^

**Figure 3:**
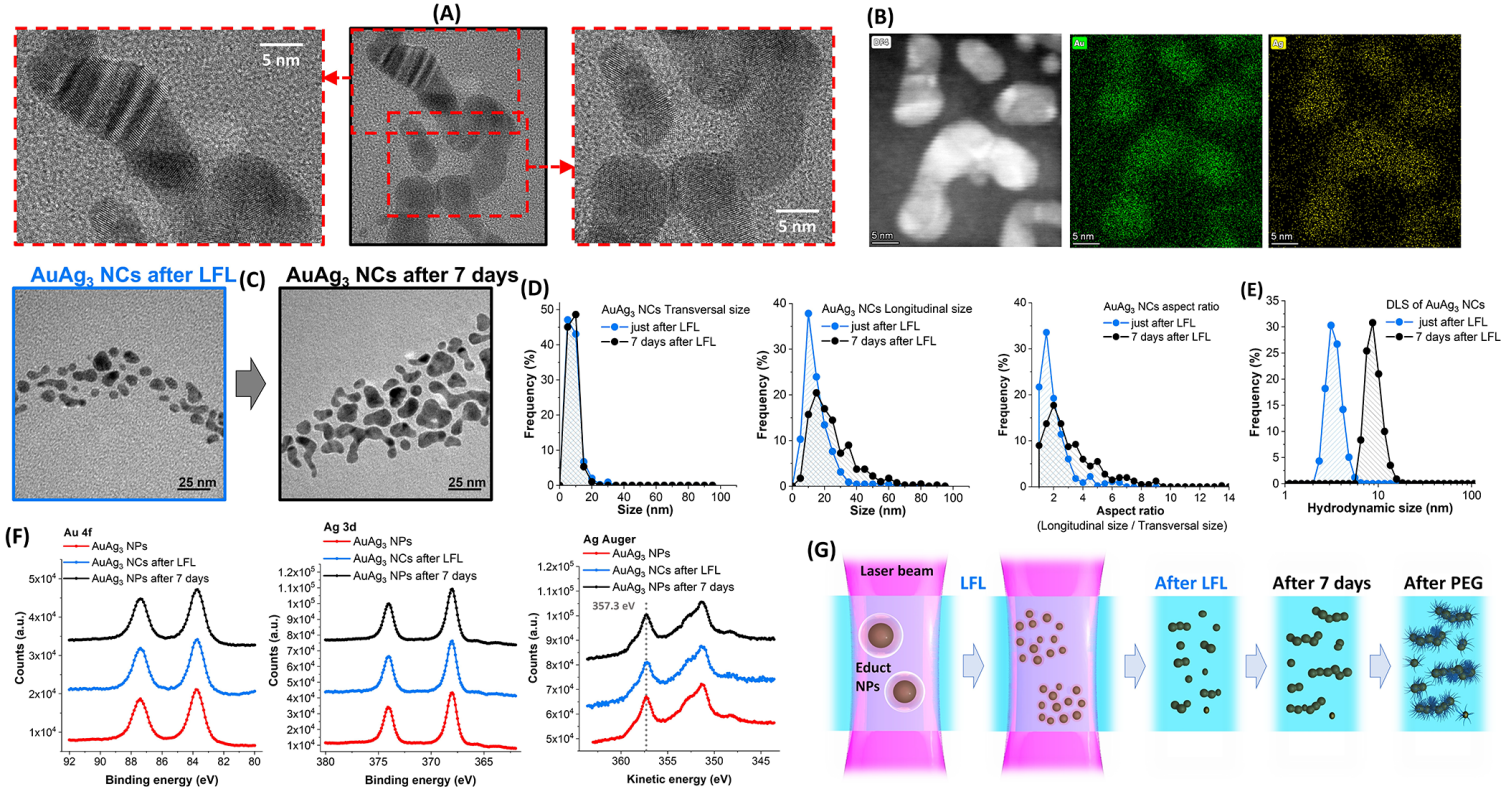
(A) HRTEM images of PEG-coated AuAg_3_ NCs. (B) EDX mapping of Au M and Ag L lines in AuAg_3_ NCs. (C) TEM images of PEG-coated AuAg_3_ NCs just after LFL and after aging for 7 days. (D) Histograms of AuAg_3_ NCs transversal and longitudinal size and of their aspect ratio. (E) DLS analysis of PEG-coated AuAg_3_ NCs colloid in water. (F) XPS analysis of Au 4f, Ag 3d, and Ag Auger lines of pristine AuAg_3_ NPs (red), AuAg_3_ NCs just after LFL (blue), and after aging for 7 days (black). (G) Sketch of Au–Ag NCs formation in three stages: photofragmentation, regrowth in anisotropic particles with low aspect ratios soon after fragmentation, and further coalescence and soldering of these particles in NCs with a higher aspect ratio. The process is concluded after PEG coating.

High-resolution TEM analysis on the AuAg_3_ NCs (Figure 3A) fully supports this mechanism, because a polycrystalline structure with single grain size equaling the transversal size of the NC was evidenced. On the contrary, the bidimensional energy dispersive X-ray (EDX) mapping confirmed the expected homogeneous chemical composition of the Au–Ag alloy (Figure 3B).

To obtain more information on the growth mechanism, AuAg_3_ NCs were coated with m-PEG-SH soon after LFL or after 7 days to interrupt the coalescence, and the two samples were analyzed with TEM (Figure 3C,D) and dynamic light scattering (DLS, Figure 3E). TEM analysis indicated that the transversal size of the NCs remains unchanged over a week, while the longitudinal size undergoes a remarkable increment, with a consequent net increase of the aspect ratio from 2.0 ± 1.0 to 3.1 ± 1.8 (Figure 3D). The hydrodynamic size measured by DLS further confirmed the growth of NCs over 7 days, although the measurement cannot be directly compared with the geometrical size assessed by TEM due to the NCs asymmetric shape and their polymeric shell. In particular, the hydrodynamic size of the AuAg_3_ NCs sample changes from 3.2 ± 0.6 nm just after LFL to 9.0 ± 1.8 nm after 7 days. Besides, X-ray photoelectron spectroscopy was performed on AuAg_3_ samples (NPs before LFL, NCs just after LFL, and NCs after 7 days of aging, all without any surface conjugation or purification) to check for any chemical transformation during LFL or aging. In all samples, the Auger parameter (725.4 eV) and the shape of the MNN Auger peak were typical of the metallic Ag^57^ (Figure 3F), excluding the presence of silver oxide or chloride. This agrees with EDX mapping, which did not evidence the presence of O or Cl in the AuAg_3_ NCs. The surface composition of the metal particles was obtained by considering the photoemission intensity of the 3d Ag peak and the 4f Au peak (Figure 3F), resulting in agreement with the nominal Ag/Au ratio in all the three samples. Overall, the XPS data do not indicate chemical transformations during the NCs formation, which is thus attributable only to the anisotropic coalescence and spontaneous soldering of the photofragmented nanocrystals into a unique nanostructure.^30,36,58^

According to this set of experimental evidence, the anisotropic growth of NCs may be divided in two stages (Figure 3G), with the first one occurring just after photofragmentation generating the initial NCs, followed by a second slower one taking place over several days in which the NCs coalesce and increase their aspect ratio. This is in agreement with previous observations of unidirectional self-assembly of metallic nanoparticles, which occurs with the fast formation of oligomers followed by their slower assembly into larger structures.^29,33,36,52,53^

### 2.3 Optical Properties

**Figure 4:**
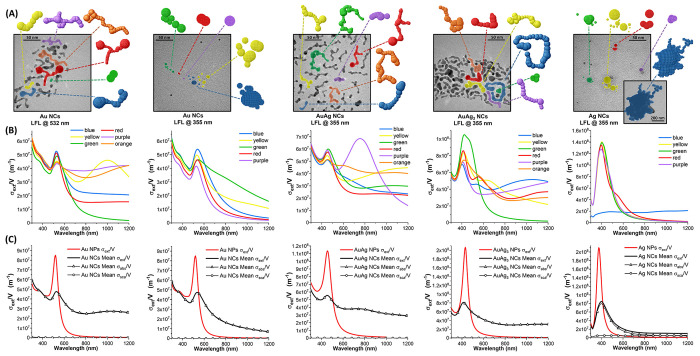
(A) DDA targets extracted from randomly chosen NCs in TEM images of each sample aged for 7 days. (B) The *σ*_ext_*/V* calculated for each of the models shown in (A), considering the orientational average to reproduce their behavior in a colloidal solution. The color of the plot indicates the corresponding target for each sample. (C) Mean extinction (*σ*_ext_*/V*, full black circles), absorption (*σ*_abs_*/V*, hollow black triangles), and scattering (*σ*_sca_*/V*, black hollow circles) resulting from the different targets for each NCs sample. The *σ*_ext_*/V* calculated for the pristine NPs is also reported for comparison (red lines).

The optical properties of NCs were investigated further by numerical calculations with the discrete dipole approximation (DDA) model.^59,60^ The DDA is a convenient tool for describing objects with any morphology and composition by a simple cubic array of polarizable dipoles, such that the accuracy of the calculated optical properties is nearly independent of object shape.^29,60^ In fact, the error of DDA is well below 10% when the interdipole spacing is small compared to the object size and the wavelengths of interest.^59,60^ Hence, a set of particles was randomly identified from the TEM images of each NCs sample aged for 7 days and transformed in an array of dipoles for the DDA calculations, as shown in Figure 4A. The extinction cross sections (*σ*_ext_) for each object of volume *V* were calculated considering the orientational average with respect to the incident electromagnetic radiation and setting water as the surrounding matrix, to reproduce the optical properties of the colloidal dispersion of NCs. In Figure 4B, the *σ*_ext_*/V* ratio is reported for each NC because it allows for a straightforward comparison of results independent of particles volume. The results are indicative of how the NCs support multiple plasmon resonances included low-energy plasmons absorbing near-infrared light. In fact, the majority of NCs in the Au–Ag samples belong to the C_1_ point group, i.e., the lowest symmetry for a single object, allowing an exceptionally high number of plasmon modes.^36,61^ This explains why the most ramified NCs exhibit flat or broad plasmon resonances in the red and NIR (black lines in Figure 4C), whose convolution originates the panchromatic absorption observed experimentally. In Figure 4C it is also reported the *σ*_ext_*/V* calculated for the pristine spherical NPs considering the TEM measured average size. Overall, the calculations reproduced all the main optical features of real NPs and NCs samples with very good agreement with the experimental results of Figure 2A in all cases. Note that, in the case of Ag NCs, it was necessary to add the contribution of a large agglomerate representative of those found during the TEM analysis (blue target in Figure 4C, weighted thrice compared to other particles), to reproduce the broadband absorption background observed in the experimental spectrum. In case of the Au NCs from LFL at 355 nm, after several unfruitful tests over multiple TEM images, it was necessary to assume that interparticle distance in each target was half of that measured from electron microscopy, to achieve a band broadening compatible with the experiment. Instead, with the interparticle distances measured from the TEM image, a low extinction in the red and NIR was systematically found.

In Figure 4C, the absorption (*σ*_abs_*/V*) and scattering (*σ*_sca_*/V*) terms are also reported for the NCs, evidencing the importance of their small transversal size to behave as a pure plasmon absorber, i.e., a plasmonic nanoparticle where the scattering cross section is negligible compared to the absorption cross section. In the specific case of Au (LFL at 532 nm), AuAg, and AuAg_3_ NCs, the *σ*_abs_*/σ*_sca_ ratio always exceeds 10^2^. In the Ag NCs case, given the sixth power dependence of the *σ*_sca_ on the particle size and the presence of a large silver agglomerate, the *σ*_abs_*/σ*_sca_ ratio of Ag NCs comes near unity in the NIR, indicating that this sample can convert only part of the extinguished NIR light into heat.

Importantly, the plots of *σ*_ext_*/V* in Figure 4B,C provide quantitative evidence of the superior plasmonic response of Ag-containing particles, which have larger extinction and absorption in the whole spectral range. The *σ*_ext_*/V* of AuAg_3_ NCs always exceeds 3.1 × 10^7^ m^–1^ in the range of our calculations, with a plasmonic peak of 8.1 × 10^7^ m^–1^ at 415 nm. The *σ*_ext_*/V* of AuAg NCs always exceeds 2.9 × 10^7^ m^–1^ with a plasmon peak of 4.6 × 10^7^ m^–1^ at 455 nm. The *σ*_ext_*/V* of Au NCs (LFL at 532 nm) is lower, with a minimum of 2.5 × 10^7^ m^–1^ at 800 nm and a plasmon peak of 4.8 × 10^7^ m^–1^ at 530 nm. The Ag NCs have the most intense plasmon peak extinction of 8.5 × 10^7^ m^–1^ at 400 nm but an absorption of only 1.0 × 10^7^ m^–1^ at 1200 nm.

It is worth noticing that, according to the numerical simulations, the panchromism occurs preferentially in long and branched NCs with homogeneous cross section (such as the orange AuAg NC and the violet Au NC) compared to shorter or less branched structures (such as the violet AuAg NC) or NC with an inhomogeneous cross section (such as the red Au NCs and the red AuAg NC). This is independent of the overall size or Au/Ag ratio of the NCs, although larger NCs are usually more branched. Because all the cross sections scale with the size of the particle, it also means that the largest and most branched NCs provide the main contribution to the panchromism of the real colloidal solutions.

### 2.4 Thermoplasmonic Properties

**Figure 5:**
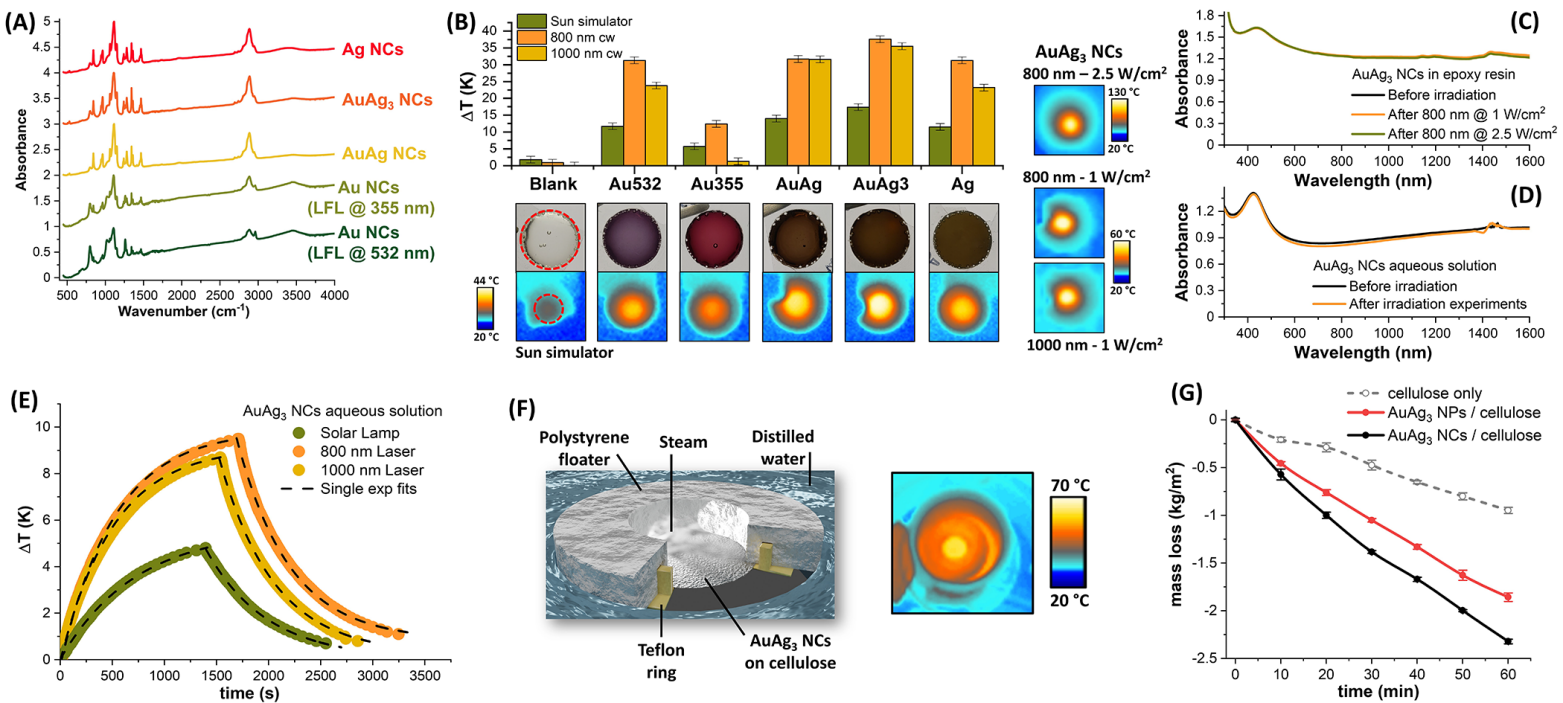
(A) FTIR spectra of the NCs samples, showing the successful coating with PEG in all cases. (B) Temperature increment (*ΔT*) measured in epoxy resins loaded with the NCs samples (pictures shown) and irradiated with a sun simulator (thermographic images shown) or cw lasers at 800 and 1000 nm. The red circles in the pictures have a diameter of 10 mm. Thermographic images for the resin with AuAg_3_ NCs while irradiated at 800 nm (1 and 2.5 W/cm^2^) and 1000 nm (1 W/cm^2^) are also shown. (C and D) Photostability tests showing UV–vis spectra of the resin (C) or the colloid (D) with AuAg_3_ NCs before and after the irradiation experiments. (E) Heating–cooling cycles of the AuAg_3_ NCs colloid with various heating sources. (F) Sketch of the solar steam generation system and thermography with the AuAg_3_ NCs on the cellulose filter and under sun simulator irradiation. (G) Water mass loss during irradiation for the cellulose substrate alone (hollow circles), loaded with the AuAg_3_ NPs (red circles) or with the AuAg_3_ NCs (black circles).

The numerical calculations indicate that AuAg_3_ NCs offer the best performances for broadband light-to-heat conversion. Hence, we tested the NCs samples with photothermal experiments in conditions of interest for practical applications. All the NCs samples are effectively functionalized with PEG simply by adding the thiolated polymer to the colloid, as demonstrated by Fourier transformed infrared (FTIR) spectroscopy of the dialyzed NCs samples (Figure 5A). Thus, taking advantage of the PEG coating and consequent easy transferability of the NCs from aqueous to CH_2_Cl_2_ solutions, the various NCs were included in a lipophilic transparent epoxy resin (Figure 5A), all at the same molar loading. Noticeably, the procedure required the drying of the NCs into a powder and their redissolution in dichloromethane, showing that the NCs can be stored as a dried powder before use. The NCs-loaded epoxy cylinders were irradiated with a sun simulator at AM 1.5, which produced the heating of the nanocomposites up to the plateaux temperatures reported in the graph of Figure 5B. In agreement with the optical properties of NCs, the highest temperature increment (*ΔT*) of 17.4 ± 0.5 °C was measured in the sample with AuAg_3_ NCs (see thermographs in Figure 5B), followed by those with AuAg (14.0 ± 0.5 °C), Au from LFL at 532 nm (11.7 ± 0.5 °C), and Ag NCs (11.5 ± 0.5 °C). The importance of a broadband absorption extending into the NIR for sunlight-to-heat conversion is evidenced by the sample with Au NCs from 355 nm, which only reached a *ΔT* of 5.7 ± 0.5 °C, not far from the transparent cylinder without NCs (1.8 ± 0.5 °C). The result of the sample with Ag NCs is explained by the intense plasmon absorption in the visible range, which, nonetheless, is not sufficient to achieve the best heating performances observed with the broadband AuAg_3_ and AuAg NCs. The heating performances were tested further with continuous wave 800 and 1000 nm laser sources (1 W/cm^2^) and the largest temperature increment was measured again for the AuAg_3_ NCs disc (Figure 5B). It is worth noticing that the *ΔT* values of samples with AuAg_3_ and AuAg NCs remain comparable both at 800 and 1000 nm, while the *ΔT* values of samples with Au NCs from 532 nm LFL and Ag NCs are sensibly lower at 1000 nm than at 800 nm, further indicating the superior panchromism of the Au–Ag alloy NCs.

The photostability of the AuAg_3_ NCs in the epoxy resin matrix was assessed by prolonged irradiation at 1 W/cm^2^ and at the maximum output laser power of 2.5 W/cm^2^, resulting in heating to a peak temperature of, respectively, 57.6 ± 1 and 127 ± 1 °C. No changes are observed in the UV–vis collected after each of the heating cycles (Figure 5C), thus showing that the NCs withstand the high local temperature of the experiments. The heating performances and photostability of AuAg_3_ NCs were further assessed in aqueous solution, by irradiation with the solar simulator and the cw laser sources at 800 and 1000 nm (1 W/cm^2^) for up to 25 min for each cycle. UV–vis spectroscopy shows that NCs completely retained the spectral features after the three heating experiments (Figure 5D), confirming their photostability also in the liquid phase. The temperature variation was monitored in real time with a thermocouple during the heating (light on) and cooling (light off) cycles, resulting in curves well-fitted with a single-exponential law, as expected for photostable compounds (Figure 5E).^62^

The positive features evidenced by AuAg_3_ NCs for sunlight-to-heat conversion motivated us to perform a proof-of-concept experiment of solar steam generation, which is an application of great contemporary interest. With the continuous population growth and the consequent environmental pollution problems, the water shortage is one of the most challenging problems of the 21st century.^9,13,15,63^ Especially for domestic use in poor regions, the supply of clean water is often prohibitive.^9,15,63^ The development of new, compact, user-friendly, and cost-effective solar steam generators is thus necessary for water purification or desalination.^9,13,15^ Thus, the NCs with their effective absorption of sunlight can act as heat spots to evaporate water. To comply with the above considerations, the experiment was conducted by keeping at maximum the simplicity and portability of the solar steam generation device, which consisted in the loading of AuAg_3_ NCs on a hydrophilic cellulose substrate. The substrate was fastened with a snap-fit Teflon ring to a floater of a white polystyrene foam put in a beaker containing deionized water, as shown in Figure 5F, and irradiated with the sun simulator at AM 1.5. Due to the hydrophilicity of cellulose,^35,63^ a thin water layer is always present above the absorbing substrate,^8^ just where the conversion of sunlight to heat takes place by the metal particles.^9^ Effective loading the cellulose with the NCs occurred just by filtration of the colloid through the substrate, without any particles surface functionalization after LFL, but by premixing with a saline buffer to reduce the electrostatic repulsion between NCs and the cellulose fibers. The same procedure was applied also to pristine AuAg_3_ NPs to make a comparison in terms of water mass loss over time. Compared to the background evaporation due to the absorption of water and cellulose in the near UV, the substrate coated with AuAg_3_ NCs provided an increment of +150% in steam formation (Figure 5G), corresponding to a steam generation rate of 2.32 ± 0.03 kg m^–2^ h^–1^ and an efficiency^64^ of 64% in our experimental conditions. Despite the simplicity of this solar steam generation device, the final result is comparable to other devices with more complex design and production protocols.^8−10,12^ The benefit in the use of AuAg_3_ NCs structure resulted in a 25% higher steam formation rate in comparison to the bare AuAg_3_ NPs. In fact, the temperature reached by the cellulose substrates after 60 min of exposure to the solar lamp, according to the thermographic measurements, resulted in 62 ± 1 °C for the AuAg_3_ NCs sample (Figure 5F), 59 ± 1 °C for the AuAg_3_ NPs sample, and 55 ± 1 °C for the bare substrate.

### 2.5 Discussion

Sunlight-driven thermoplasmonic applications demand for a set of requisites that are not easily satisfied by conventional plasmonic nanostructures.^3,11,15,25^ The solar spectrum extends from the near UV to the NIR, with 87.7% of energy comprised in the 350–1350 nm range and the 52.4% at wavelengths >700 nm.^11,13,14^ Thus, panchromatic absorption in this wide range is a first important requisite, typically difficult to achieve in noble metal nanostructures without a simultaneous increase of size and scattering cross section.^3,11,24,25^ Photostability and chemical stability are other important features often limiting the exploitation of anisotropic metal nanoparticles obtained by chemical reduction with templating agents.^13,25,37^ This is due to a generally high surface energy and the tendency to reshaping into compact spheroidal morphologies, either in the dark or at low illumination intensity.^24,25,35,65^ More inert plasmonic materials, like nitrides, have lower absorption cross section per unit volume compared to noble metals, and are seldom processable as a colloidal solution, as desirable for inclusion in matrixes and substrates.^3,9,24,66^ They also do not benefit of the easy surface chemistry of noble metals, which are functionalizable in one step with thiolated molecules.^1,24,35^ The ability to conjugate metal nanoparticles with functional organic molecules is crucial for maintaining colloidal stability in complex liquid environments such as electrolyte solutions, biological fluids, or organic solvents.^1,24,36^ Surface functionalization is key also for the addition of selectivity versus target chemical species and the formation of surface patterns or integration in specific matrixes.^1,24,35,36^

**Figure 6:**
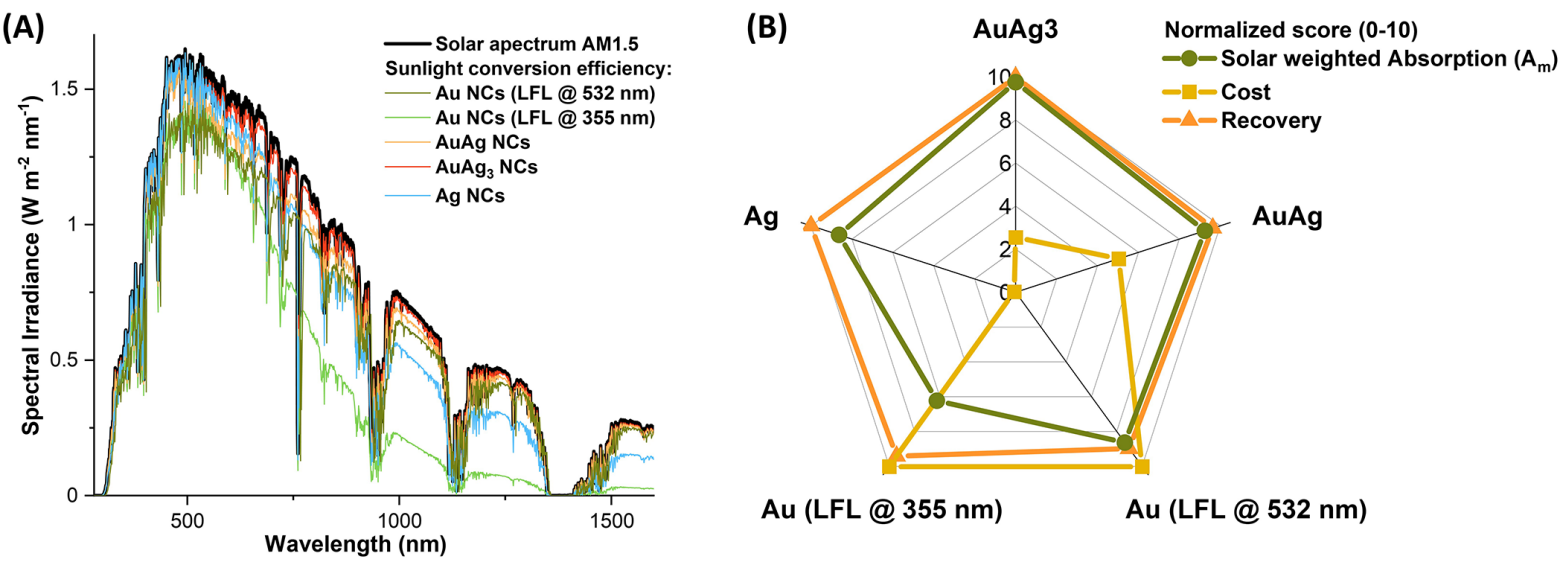
(A) Comparison of the solar spectrum irradiance (black line) and the absorbed spectral irradiance for the five NCs samples. (B) Radar graph summarizing the solar weighted absorption coefficient (*A*_m_), the cost and the recovery yield for the different NCs. For the sake of comparison, the parameters are reported on a scale from 10 to 0, where 10 means the maximization of the parameter.

The NCs satisfy well the requisites of panchromism, negligible scattering, photostability, colloidal stability, surface functionalization and processability, clean surface, and scalable synthetic procedure. On the contrary, the cost of noble metals like gold is an issue for large scale applications,^9,13,25,35,39^ but it can be lowered of 140 times per unit molar volume by resorting to silver. The sunlight to heat conversion efficiency can be properly quantified with the absorbed spectral irradiance (*A*_S_)^11^
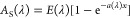
1and the solar weighted absorption coefficient (*A*_m_)
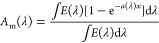
2where *E*(*λ*) is the spectral distribution of the solar intensity and *x* is the thickness of the absorbing layer with linear absorption coefficient *a*(*λ*). The plot of *A*_S_ for an absorbing layer of 1 cm and a 1 mg/mL concentration in metal atoms is reported in Figure 6A for the five NCs, definitively evidencing the superior performances of Au–Ag alloy NCs. The solar weighted absorption coefficient of the five types of NCs (integrated in the range 280–1600 nm) is reported in the radar graph of Figure 6B, also with their net cost and the material recovery during synthesis. The recovery from educt material into products often is a limitation to the cost affordability of chemical procedures, but it is nearly 100% in laser-assisted procedures, as far as the colloids maintain an appreciable stability.^29,67^ The radar graph evidences how the maximization of all these three parameters is challenging for noble metal NCs, except for the Au–Ag alloys, which reaches the best scores. In particular, the AuAg_3_ NCs perform well thanks to the combination of optical properties and cost lowering due to the silver component. This positive set of performances has been demonstrated further by the realization of the cellulose-based solar-steam generator with a distillation ability of 1 kg/h of water under AM1.5 irradiance at the cost of only few euros. The device has a minimalist design and was made possible by the simple embedding of AuAg_3_ NCs onto a cheap, hydrophilic, flexible, and foldable substrate like cellulose.^35,63^

Besides, the appealing photothermal performances, the possibility to coat their surface with biological molecules and the presence of silver make Au–Ag NCs also promising for antimicrobial applications on the basis of synergistic thermoplasmonic and biochemical effects. Alternatively, the electric conductivity of the Au–Ag alloy opens the way to the implementation of the NCs in light-triggered photothermoelectric devices and nanocomposites.

Concerning the laser-assisted synthetic procedure, the resorting to Au–Ag alloys also provided a net advantage over pure Au and Ag NPs. During LFL at 355 nm, the photofragmented Au–Ag alloy nanocrystals spontaneously continue their growth by unidirectional assembly in solution, because of the balance between the electrostatic repulsive force and the attractive dipolar interactions in the colloidal system.^36,52,53^ It has been calculated that the repulsive forces have lower intensity along the axis of an elongated particle in a colloidal solution, than on its sides.^52,53^ This promotes the coalescence and unidirectional growth of metal NPs in a colloidal solution, when the surface of the NPs is not stabilized against aggregation. After aggregation, the soldering of the interface is possible due to the high mobility of metal atoms on surfaces with nanoscale curvatures and in aqueous solutions of NaCl. However, the stability of the colloidal system is dramatically altered by the introduction of a steric stabilizer, which chemically bind to surface metal atoms, such as thiolated PEG. In fact, the coalescence process was inhibited by PEG addition, in agreement with what was observed previously with Au NCs.^36^

The formation of NCs has not occurred with Ag or Au NPs irradiated at 355 nm, which yielded only spherical or slightly spheroidal nanocrystals. It has been shown that a cluster cloud of atoms is involved in colloidal metal NPs prenucleation, nucleation, and maturation and this supersaturated cloud condenses around crystalline seeds oscillating between amorphous and crystalline states.^68^ Besides, molecular dynamics simulation^69,70^ and in situ TEM analysis^71^ both indicated that crystalline metallic seeds form together with atomic vapors during laser photofragmentation, and these vapors may also promote the growth of asymmetric particles already on the nanosecond time scale.^69^ In the LFL case, photofragmentation is a single step and instantaneous process^69,72,73^ that occurred in all samples irradiated at 355 nm, after which the photochemistry of silver and gold atoms synergically contributed to the generation of NCs after photofragmentation.

The single-step (i.e., single-pulse) nature of the photofragmentation process is supported by the LFL optimization experiments performed at different NPs feeding rates between 0.25 and 0.13 mL/min or at different concentrations between 0.75 and 0.35 mM in metal atoms. The NPs concentration produced almost no effects on the panchromism of the NCs, although it has been reported that incomplete photofragmentation should occur when the concentration of the initial NPs exceeds a threshold that depends on laser pulse wavelength, duration, energy, and optical path.^67,73^ Instead, the feeding rate must be high enough to avoid irradiation of the NPs with multiple pulses. At a low feeding rate of 0.13 mL/min, an increase of the main plasmon peak was observed, which is indicative of the presence of spherical particles. This is attributed to the reshaping of the initial asymmetric structures into spherical or spheroidal ones, due to the absorption of multiple pulses. A similar reshaping effect has been reported several times in the literature, particularly with nanorods.^74^ Indeed, this opens new perspectives in the laser irradiation of the NCs as a strategy to adjust their morphology and tune their optical properties. For instance, by a “spectral hole-burning” experiment in which only the NCs absorbing at a specific wavelength are photodisintegrated or photomelted, as demonstrated by El-Sayed et al.^74^

## 3 Conclusion

With more than 50% of solar energy being emitted at wavelengths longer than 700 nm, efficient sunlight-driven photothermal applications are only possible with broadband absorbers. Noble metal nanoparticles exhibit intense and tunable plasmon absorptions that either cover a limited spectral range or result in the prevalence of scattering over absorption. Here, we showed that it is possible to use laser light to harvest solar light by the realization of Au–Ag alloy NCs with optimal features for sunlight-driven thermoplasmonics. These NCs have broadband plasmon absorption extending from the visible to the near-infrared, even beyond 1350 nm, with cross sections larger than Au equivalents and an absorption to scattering ratio exceeding 10^2^, as estimated by numerical calculations. The free surface of Au–Ag alloy NCs allows for the functionalization with thiolated molecules like PEG, enabling nancomposite formation by inclusion in lipophilic epoxy resins, as well as strong interaction with green and hydrophilic substrates like cellulose. The Au–Ag NCs show efficient thermoplasmonic properties and excellent photostability under illumination with a solar simulator as well as with continuous wave laser sources at 800 and 1000 nm, both in nanocomposites and as a colloidal dispersion. Besides, a solar-steam generation device with low-cost, ultrasimple design but very good distillation power of 2.32 ± 0.03 kg m^–2^ h^–1^ was demonstrated with the Au–Ag NCs. Importantly, the Au–Ag NCs are produced with a self-standing, green, and scalable methodology relying on pulsed laser fragmentation in liquid under continuous flux of pristine Au–Ag nanoparticles produced by laser ablation in liquid.

With their optimized panchromism, the thermoplasmonic performances, the green synthetic procedure, and the other set of positive features, the Au–Ag NCs mark a contribution to the development of environmentally friendly devices for sunlight-driven photothermal applications of practical utility in a sustainable world.

## 4 Experimental Methods

### Synthesis

Au, Ag, AuAg, and AuAg_3_ NPs were obtained by LAL using solid targets (6 mm in diameter) with the respective composition dipped 0.2 mM NaCl (≥99.5%, Fluka) solutions in distilled water. Laser pulses at 1064 nm (6 ns, 50 Hz) of a Q-switched Nd:YAG laser were focused with a f 100 mm lens up to a fluence of 8 J/cm^2^. The ablated target area was set to a circular Archimedean spiral with maximum diameter 5 mm, completed in 200 s, by mounting the cell on a motorized XY scanning stage (Standa) managed with a two-axis stepper, a DC motor controller, and a custom-made LabView program.

Au, Ag, AuAg, and AuAg_3_ NCs were obtained by LFL of the corresponding NPs solutions diluted 1:1 with ethanol (HPLC grade, Sigma-Aldrich) and set to a final concentration of metal atoms in the 0.5–0.6 mM range. The liquid was fluxed through a glass channel (diameter of 1.5 mm) at a velocity of 0.2 mL/min. Laser pulses at either 532 or 355 nm (6 ns, 10 Hz) from the duplicate or triplicate of a Q-switched Nd:YAG laser were focused on the glass channel at a final fluence of 1200 mJ/cm^2^. For process optimization with Au–Ag alloy NPs, the feeding rate was tested between 0.25 and 0.13 mL/min and NPs concentration between 0.75 and 0.35 mM in metal atoms.

The aging of NCs was performed in the dark, at room temperature in glass vials. Surface functionalization was performed by room temperature incubation of the NCs solution with thiolated methoxy poly(ethylene glycol) (m-PEG-SH, 6000 Da, Sigma-Aldrich) for 90 min. Excess PEG was removed by dialysis with Vivaspin 10 kDa concentration membranes at 800 rcf followed by three washing cycles with distilled water.

The epoxy resin nanocomposites were obtained from NCs dissolved in CH_2_Cl_2_ (HPLC grade, Sigma-Aldrich). Equal volumes (2 mL) of the NCs aqueous solutions, all at the same molar concentration of 0.5 mM in metal atoms, were first dried in air at 30 °C and then redissolved in CH_2_Cl_2_ at the same initial concentration before mixing 100 μL with 250 μL of the bicomponent epoxy resin. Finally, the mixture was poured into a Teflon mold (10 mm in diameter) coated with a Kapton film for overnight.

To support the metal particles on the cellulose substrate, the AuAg_3_ NPs or AuAg_3_ NCs colloids in water were mixed with 20 mM HEPES (2-[4-(2-hydroxyethyl)piperazin-1-yl]ethanesulfonic acid) buffer solution and then syringe filtered through hydrophilic cellulose acetate filters (0.45 μm cutoff, 25 mm filter diameter, VWR International).

The ratio between NPs solution and HEPES buffer was set to 1:0.4 vol/vol to achieve quantitative particles sticking on the filter after two filtrations, while avoiding precipitation in the liquid solution. For each substrate, 35 mL of colloid at a concentration of 0.06 mg/mL was mixed with 15 mL of 20 mM HEPES solution.

### Characterization

UV–visible–NIR spectroscopy was performed with a JASCO V-770 spectrometer using 2 mm optical path quartz cells. TEM analysis was performed with a FEI Tecnai G2 12 transmission electron microscope operating at 100 kV and equipped with a TVIPS CCD camera. Samples were prepared by evaporating the colloids on a copper grid coated with an amorphous carbon holey film. Statistics considered >500 nanoparticles for each sample, using the ImageJ software. HRTEM and EDX analysis were performed with a Talos F200S (Thermofisher Scientific) instrument operating at 200 kV. Elemental maps were obtained from the Au M and Ag L lines.

FTIR measurements were performed with a PerkinElmer 1720X spectrometer. Samples were obtained by evaporating the solvent and depositing the NPs powder on a KBr substrate. DLS measurements were performed with a Malvern Zetasizer Nano ZS in ZEN0040 cells.

XPS analysis was performed at room temperature using normal emission geometry with a modified VG ESCALAB MKII (Vacuum generators, Hastings, England) equipped with a twin (Mg/Al) anode X-ray source, a sputter gun, and a hemispherical electrostatic analyzer with a five-channel detector. As an excitation source, we used Mg Kα radiation (1253.6 eV). The sample was obtained by dropwise deposition of a AuAg_3_ NPs or NCs dispersions on a Cu sample holder and drying at room temperature. Surface composition was obtained from 3d Ag and 4f Au peaks using sensitivity factors calculated on the basis of the photoemission cross sections reported in ref (75) and inelastic electron mean free path determined by the TPP2 algorithm.^76^

### Numerical Calculations

Numerical calculations of the optical properties with the DDA method were performed with the DDSCAT 7.3 code.^59^ The SPHERES_N routine was exploited to reproduce the same size and geometric position of the particles or group of particles in the TEM pictures, by creating each target ad hoc. The number of dipoles (*N*) was set between 10^4^ and 10^5^ to have an interdipole spacing much lower than the particles size and the shortest wavelength considered, as required to obtain an error well below 10% on the computed cross sections for metal particles in the 2–200 nm size range.^59,60^ All the calculations considered the arithmetic average over two orthogonal polarization directions and 27 sets of Euler angles of rotation of the target with respect to the incident plane wave (i.e., a total of 54 different orientations for each target) to simulate the random orientation of particles in the liquid solution.

The experimentally measured complex optical constants of Au, Ag, AuAg, and AuAg_3_ were obtained from refractiveindex.org or as described in refs (36, 38, and 40). Calculations were performed in the 300–1200 nm range, which was the only one compatible with all the available optical constants. All optical constants were corrected for the intrinsic size effects according to what are described in refs (2, 36, 38, 40, and 55). The water solvent was accounted by setting the refractive index of the nonabsorbing matrix to 1.334.

### Photothermal Experiments

Photothermal heating experiments with the sunlight spectrum were performed irradiating all the samples surface with an AM1.5 sun simulator (LOT-Quantum Design solar simulator AM 1.5 G) at a distance of 10 cm. The irradiation at 800 and 1000 nm were carried out with a Spectra-Physics 3900s titanium/sapphire continuous wave tunable laser pumped by a Coherent Verdi G7 OPSL laser. The laser power was set at 200 mW for each wavelength, and the laser spot diameter was 5 mm.

A thermal camera model FLIR E5 was used to capture the calibrated digital thermographic infrared images of the heated samples. The temperature in the liquid samples was also monitored with a K-type thermocouple dipped in a dark region of the cuvette.

Solar steam generation experiments were performed in a beaker containing 50 mL of deionized water and a floating device with the cellulose substrate. The device consisted in a holed circular polystyrene foam (40 mm external diameter, 14 mm internal diameter) and a snap-fit Teflon support to fasten the cellulose substrate. The beaker was irradiated with the light of the AM1.5 sun simulator and the accessory for normal incidence, at a distance of 5 cm and at room temperature. The liquid mass loss with the bare, AuAg_3_ NPs, or AuAg_3_ NCs loaded cellulose substrates was measured in triplicate with a KERN PLE-N digital balance over 60 min. The local temperature was registered with the FLIR E5 thermocamera.
